# Valorization Strategy for Leather Waste as Filler for High-Density Polyethylene Composites: Analysis of the Thermal Stability, Insulation Properties and Chromium Leaching

**DOI:** 10.3390/polym13193313

**Published:** 2021-09-28

**Authors:** Eylem Kiliç, Helena Oliver-Ortega, Quim Tarrés, Marc Delgado-Aguilar, Pere Fullana-i-Palmer, Rita Puig

**Affiliations:** 1 Material Science and Nanotechnology Engineering Department, Usak University, 64200 Usak, Turkey; 2 LEPAMAP-PRODIS Research Group, University of Girona, 17003 Girona, Spain; helena.oliver@udg.edu (H.O.-O.); joaquimagusti.tarres@udg.edu (Q.T.); m.delgado@udg.edu (M.D.-A.); 3 UNESCO Chair in Life Cycle and Climate Change ESCI-UPF, Universitat Pompeu Fabra, 08003 Barcelona, Spain; pere.fullana@esci.upf.edu; 4 ABBU Research Group, Department of Computer Science and Industrial Engineering, Universitat de Lleida (UdL), 08700 Igualada, Spain; rita.puig@udl.cat

**Keywords:** leather waste, HDPE, composites, thermal properties, leaching

## Abstract

Leather waste (BF) and high-density polyethylene (HDPE) were compounded in a lab scale internal mixer and processed by means of injection molding. In this study, leather waste and HDPE composites were characterized by instrumental techniques such as differential scanning calorimetry (DSC), thermo-gravimetric Analysis (TGA), and Fourier transform infrared spectroscopy (FTIR). Physical integrity of composites against chemical exposure and chromium-leaching properties of the composites were also investigated. This study shows that the incorporation of 30% leather waste fiber into HDPE composites decreases the thermal conductivity of the composite samples by 17% in comparison to that of neat HDPE samples. Composites showed no thermal degradation during processing cycle. Strong interfacial bonding between leather waste and polymer results in comparable low-leachate levels to maximum allowed concentration for nonhazardous waste, and good chemical resistance properties. The BF/HDPE composites could be a promising low-cost alternative in industrial application areas of HDPE, where high-mechanical strength and low-thermal conductivity is required.

## 1. Introduction

The recent increasing restrictions on traditional waste treatments and the growing concerns about overexploitation of natural resources, connected with severe environmental impacts, are promoting the implementation of a circular economy. The aim of a circular economy is to minimize resource use and the associated environmental pressures by improving waste management practices through recycling, reuse of products, avoiding waste, and turning unavoidable waste into a resource. The critical discussions about implementing a circular economy and a sustainable use of natural resources led industries to focus on developing a more resource-efficient approach in their production systems.

Composite industry is one of the prominent growing sectors due to widened demand for high-performance materials and their vast usage in engineering applications, especially in the aerospace and automotive industries [1]. The industry is highly innovative and consistently searches for new material types to achieve favorable result for different applications, considering their physical properties, as well as economic and environmental performances. The considerations regarding transition towards a circular economy led composite industry to focus on renewable raw materials, which enhanced the interest in replacing one or all the phases of composites with more environmentally friendly components, such as natural fibers. Natural fibers provide a number of advantages compared to that of synthetic fibers, due to their abundance, availability, low-cost, and sustainability [2].

In the concept of efficient use of natural resources, the use of natural fibers derived from industrial and agricultural wastes as reinforcement material in thermoplastic polymer composites was a prominent direction of research, both in terms of valorization of agricultural wastes and recycling of industrial solid waste. Over the years, substantial research took place to develop and improve properties of natural fiber reinforced composites by using waste fibers from different plant sources (barley, peanut shell, olive hut, bamboo, etc.) [3,4,5,6]. On the other hand, comparatively fewer studies were reported using natural fiber wastes from animal origin, such as chicken feather, wool waste, and [7,8,9] leather waste generated in leather processing [10,11,12].

Leather waste is one of the major sources of animal-based collagen fibers, which provide mechanical strength and flexibility to the skin. Throughout tanning process, collagen fibers are cross-linked with tanning agents and raw skins/hides are converted into leather. Only the 20% of the rawhide is transformed into leather final product, and the 45% of raw hide is discarded in the form of nontanned and tanned collagenous waste [13]. Among the tanned collagenous wastes, the chromium containing solid waste, generated when the finished leather is subjected to abrasion process to provide a uniform feature, is referred as buffing dust (BF). BF is a micro fined powder of cross-linked collagen fibril, containing chromium, synthetic fat, and other chemicals. For every tone of skin or hide processed, approximately 2–6 kg of BF is generated as solid waste.

Contrarily to other types of leather industry solid wastes, the treatment of BF is difficult and of great environmental concern. Exposure to its particles may cause respiratory tract irritation in humans, as they contain trivalent chromium along with organic and inorganic compounds [14,15]. BF wastes are either incinerated or disposed to landfill sites, which account for serious environmental impacts and economic loss due to overpriced and limited available landfill sites [16]. Researchers made various attempts for finding beneficial uses of this waste and utilized BF waste as fillers for rubber [17], polylactic acid [11], and polycaprolactone [18], polystyrene [19], and epoxy-polymer [20] matrices to produce low-cost composite materials for various industrial applications (footwear industry, bags, suitcases, etc.) [18,21]. The literature shows that a great number and type of fillers were incorporated into HDPE to produce inexpensive HDPE composites with reduced environmental impact and enhanced properties [22], considering the wide application area and its contribution to worldwide plastic consumption [23]. Successful utilization of BF (30%) as reinforcement material in HDPE matrix resulted in superior mechanical properties, particularly improved impact resistance, and provided recyclability up to 5 recycling cycles due to strong fibrillar-like interfacial interactions between leather waste and polymer. This well-bonded system was observed and supported by SEM images in a previously published paper by our research team [24,25]. However, the literature related to thermal characteristics and chemical resistance is incipient, as well as the chromium leaching from the composites and the presence of Cr(VI) in the leachate.

For all the above, the present work aims at determining the impact of BF on the thermal properties of HDPE, including thermogravimetry, calorimetry and insulation, the chemical resistance and the Cr(VI) content of the leachate from composites after inducing the leaching process, which provides useful information of the end-of-life of the obtained materials.

## 2. Materials and Methods

### 2.1. Materials

Leather buffing dust waste (BF) with a particle size of the leather fiber, in the range of (0.3 mm diameter and 0.09 mm length) was provided from a sheepskin processing leather factory in Turkey. Leather waste fibers were used without any additional or prior treatment such as milling, grinding, or chemical treatment. The characteristics of BF are listed in Table 1 [25]. HDPE Braskem 7252 was supplied by Nexeo Solutions (Sao Paulo, Brazil) and was used as thermoplastic matrix.

### 2.2. Method

#### 2.2.1. Compounding and Injection Molding of Composite Specimens

HDPE pellets and BF were dried at 80 °C, before processing, to assure very low water content. Composite samples with leather fiber content from 20 to 50 wt% were prepared by using a Brabender® (Brabender GmbH & Co KG, Duisburg, Germany) internal mixing machine. Materials were fed to mixing machine and compounded at 80 rpm speed for 10 min at 170 °C. Thereafter, the composite blends were granulated into pellets using a blade mill equipped with a 10 mm mesh strainer and kept in oven (Dycometal, Barcelona, Spain) at 80 °C until further use to prevent moisture absorption. The obtained pellets were injection molded using Meteor 40 injection-molding machine (Mateu & Sole, Barcelona, Spain) to produce dog bone composite test specimens complying with ASTM D3641 specifications. 

The composite specimens were conditioned in a climatic chamber (Dycometal, Barcelona, Spain) at 23 °C and 50 % relative humidity for 48 h before the tests were performed, according to the ASTM D618-13 (2013) and ASTM D638-14 (2014) standards. 

#### 2.2.2. Thermogravimetric Analysis (TGA)

Thermogravimetrical analysis (TGA) was performed using a Mettler Toledo SDTA851 thermobalance (Mettler Toledo, L’Hospitalet de Llobregat, Spain). The samples were heated from 30 °C to 700 °C at a heating rate of 10 °C/min. The test was performed in an inert atmosphere with a nitrogen flow rate of 40 mL/min.

#### 2.2.3. Differential Scanning Calorimetry (DSC)

Differential scanning calorimetry was performed using a Mettler Toledo DSC822^e^ thermal analyzer (Mettler Toledo, L’Hospitalet de Llobregat, Spain) to evaluate the influence of the incorporated leather fibers on the thermal transitions and on the crystallinity degree of matrix following ASTM E1269.01 standard specifications. The samples were initially heated from 30 to 220 °C for the first heating scan to erase their thermal history. Afterwards, cooling was performed, and the samples were heated again using the same temperature range. All runs were performed at heating or cooling rates of 10 °C/min under 40 mL/min flow of nitrogen atmosphere.

#### 2.2.4. Thermal Conductivity

Thermal conductivity of composite samples was analyzed by means of an insulated chamber equipped with four thermocouples and an electrical heating system (Honeywell, Charlotte, NC, United States) for air. This method is based on the UNE-EN 8990:1999, where the thermal transmission properties of samples are determined in steady state. The samples were positioned in the middle, dividing the chamber into two chambers (the so-called hot and the cold chambers), maintained at different temperatures in steady-state conditions. 

The thermocouples were placed at the hot chamber, and the thermal resistance of the samples was obtained by measuring the power required to keep the hot chamber at constant temperature and the temperature difference between the hot and the cold chambers. The temperature of hot side of the composite samples, the cold side of the composite samples, and the cold chamber was recorded (every minute) by means of a data acquisition system. 

Thermal conductivity, was calculated using Equation (1):

(1) $$\lambda =\frac{e.U.\left(Th-Tc\right)}{Ths-Tcs}$$

where *λ* is the thermal conductivity (W/m °C), *e* is the thickness of the composite sample (m), *U* is the global heat transfer coefficient (W/m^2^ °C), and *Th*, *Tc*, *Ths*, and *Tcs* are the temperatures of the hot chamber (*Th*), cold chamber (*Tc*), hot side (*Ths*), and cold side (*Tcs*), respectively, and all in °C. In addition to this, the *U* was calculated from Equation (2):

(2) $$U=\frac{\left(Th-Ths\right)}{\left(Th-Tc\right).\;Rsi}$$

where *Rsi* is the inner surface thermal resistance (m^2^ °C /W) and accounts for 0.13 m^2^ °C /W, according to UNE-EN 8990:1999 for vertical enclosures. The temperatures of the two chambers are maintained constant and steady state conditions were kept.

#### 2.2.5. Fourier Transform Infrared (FT-IR) Spectroscopy

Fourier Transformed infrared (FT-IR) measurements of BF waste, HDPE polymer and composites containing 30 wt% BF was carried out to determine the formation and changes in the functional groups of samples. IR spectra were obtained in the frequency range of 4000 and 400 cm^−1^ using a Bruker Alpha FT-IR spectrometer (FT-IR) (Bruker, Madrid, Spain).

#### 2.2.6. Chemical Resistance Test of Composites

Composite samples were cut into 0.5 × 0.5 cm^2^ pieces and placed into specimen jar bottles, and then immersed into various chemicals (Merck, Darmstadt, Germany) such as acetone, methanol, sodium hydroxide solution (NaOH, 30% w/w in aqueous solution), hydrogen peroxide (H_2_O_2_, 50% w/w in aqueous solution), ethyl acetate, and acetonitrile, and left for 24 h to evaluate sample’s resistance to each chemical. Variation at weight and change of visual appearance was examined following 24 h of contact with chemicals. 

#### 2.2.7. Chromium Leaching and Cr (VI) Analysis of Composites

To determine the extractable chromium leached from the composite sample, chromium leaching tests of composite samples incorporated with 30% of BF were performed according to ISO 17072. Extractable chromium in composite samples was determined by carrying out extraction with an acid artificial-perspiration solution and subsequent determination with inductively coupled plasma optical emission spectrometry. Solutions leached from composites samples were also analyzed for the determination of chromium (VI) content under defined conditions, in accordance with international standard ISO 17075-2.

## 3. Results and Discussion

### 3.1. Thermogravimetric Analysis (TGA)

**Figure 1:**
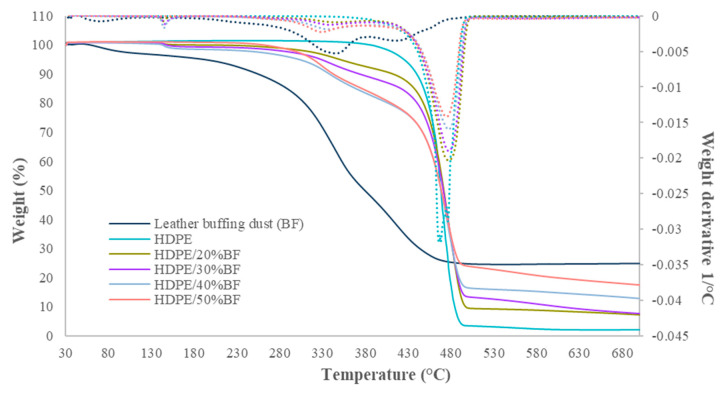
TGA curves and first derivate of TGA curve for neat HDPE, leather waste, and 20 to 50 wt% BF reinforced HDPE composite materials.

The thermal stability of neat HDPE, leather BF waste, and 20, 30, 40, and 50 wt% BF reinforced HDPE composites were analyzed with TGA. The results of the TGA assays and the first derivate (DTGA) are represented in Figure 1.

The TGA figure (Figure 1) shows the mass loss against temperature, while the DTGA allows to understand better the degradation process produced for BF and BF composites sample. The BF showed a degradation mainly between 30 to 500 °C, with different steps. In the DTGA, it was possible to differentiate at least 5 different processes, with the higher degradation rate around 70, 235, 345, 415, and 455 °C. Figure S1 show the different processes in the BF. The processes at 235 and 455 °C are overlapped by the others, but the change in the curve trend seems to indicate the change in the degradation, which could be devoted with another process starting. 

The BF weight loss at relatively low temperatures (T < 100 °C) is due to elimination of absorbed water by leather fibers. The BF waste is rich in collagenous, a protein which tend to have a high interaction with water. The change in the mass loss tendency and the DTGA confirms the apparition of another degradation process, probably related with the protein denaturalization. The weight loss that takes place between 150 and 300 °C, with the higher rate around 235 °C, can be attributed to volatile compounds such as oil and low molecular weight greases existing in the leather fibers [26,27]. The third degradation process, that starts around 300 °C with a higher rate, can be assigned to the decomposition of collagen protein into intermediate compounds, in line with the previously reported studies [18,26]. Around 500 °C, the degradation processes seem to be finished and no further weight loss is produced. Similar degradation behavior for leather fibers was observed in studies by Ambrosio et al. [27]. The residue at 700 °C accounts for a 33 wt%. 

The TGA analysis of neat HDPE matrix shows a thermally stable material up to 380 °C. Then, HDPE presents a narrow degradation step between 400–490 °C. A total of 2 wt% residue is obtained at 700 °C. In the case of the composite material, the degradation steps of the leather BF became more appreciable as we increase the amount of leather BF in the composite materials. A small and fast degradation occurred around 150 °C, which could correspond to the first degradation step of BF (excluding the water loss). Besides, the mass loss in that process is small. Around 300 °C, a high loss of mass is observed, which coincides with the second and higher degradation step of BF. The next degradation of the BF, around 415 °C, seems to be overlapped with the HDPE degradation, and a single process is appreciated in composites materials. 

The onset degradation temperatures for neat HDPE and composites are given in Table 2, as well as remaining mass at 700 °C. Onset degradation temperature, considered in the range of 10% mass loss, was affected by the filler content at range from 333 to 403 °C. Composite material reinforced with 40% of fiber showed slightly lower temperatures than the expected. It could be related with the presence of BF agglomerates in the tested samples, which led to a reduction of the onset temperatures. The temperature at 25% and 50% of mass loss is close to the 50% composites material, which is in agreement with a lower polymer volume and higher BF content in the tested sample. Besides, all samples have a similar trend and charred residue amount at 700 °C increased with the increased additive amount of BF content.

Besides, and although the BF reduced the thermal stability of the composite materials, taking into consideration the processing temperature of neat HDPE, which is around 180–200 °C, composites filled with BF can be processed without confronting a thermal degradation in the processing machines. The 30 wt% reinforced composite maintained nearly 99% of its weight at this temperature; hence, no degradation is likely to take place during the short processing cycle of the composite.

### 3.2. Differential Scanning Calorimetry (DSC)

**Figure 2:**
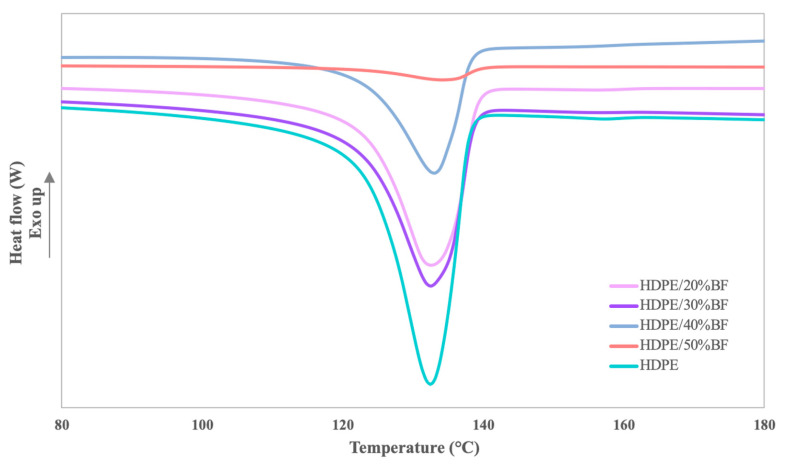
DSC thermographs of melting point of neat HDPE and 20 to 50 wt% BF-reinforced composite materials.

Although the thermal stability of the composite materials in the working and manufacture range seems to not be affected by the incorporation of BF as reinforcing filler, the main transitions temperatures of HDPE could be affected. The melting and crystallization behavior of the HDPE matrix and the BF reinforced composites were studied by means of DSC. Samples were subjected to a first heat to erase the thermal history of the materials. The thermograph obtained during the second heating of HDPE and its composites shows a single endothermic process corresponding to the melting of the HDPE matrix. An amplified thermograph of the process is shown in Figure 2.

The intensity and the enthalpy of the peak is reduced during the melting process when the fiber load is increased in the composite. Nonetheless, it is due to the lower polymer content in the composite material. The results related with the filler quantity are shown in Table 3. Thus, all the materials showed a similar melting behavior with slight increase of the melting temperature when increasing the BF content. Nevertheless, the maximum increment observed was 2 °C and it is not representative; it is in concordance with the observed in the crystallinity degree of the polymer, calculated considering the enthalpy of the 100% crystallized HDPE of 293 J/g [27,28]. Only the 40 wt%-reinforced composite showed differences in the melting enthalpy, however it could be devoted with aggregates in the sample, as above mentioned in the TGA. The incorrect dispersion of the fibers and the presence of agglomerates could affect the crystallization process as it disturbs the polymer process. Moreover, if a higher fiber content than the expected is presence in the sample it led to an incorrect enthalpy and crystallinity values because the polymer weight is lower. Thus, the presence of the BF, when it is correctly dispersed, does not seem to have any negative impact in the melting process.

Regarding the cooling process, the HDPE and composites materials showed also one single exothermal process related with the crystallization process of the HDPE. The peak temperatures are shown in Table 3. In general, the composite materials showed a slightly lower crystallization temperature, being the lowest at 50% of reinforced composite. The lower temperature could be associated with an easily crystallization process due to the presence of the BF fibers. Nonetheless, the differences are quite small to be considered to have a huge impact in the crystallization process.

**Figure 3:**
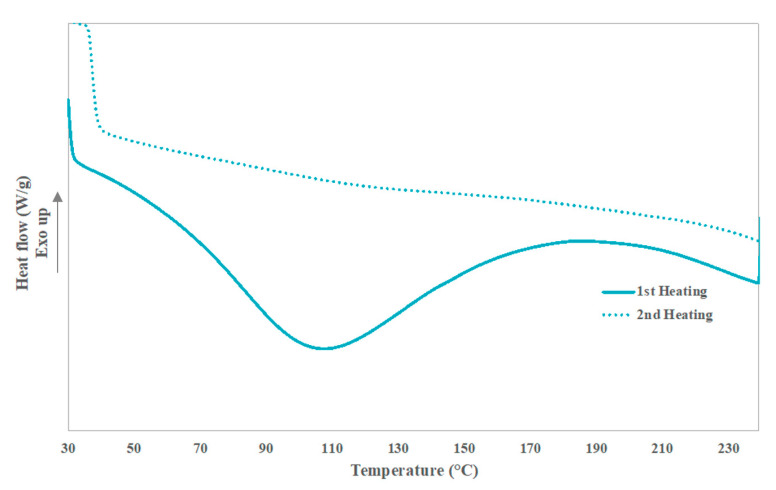
DSC thermograph of buffing dust waste.

The DSC thermograph of the 1st and the 2nd heat of the BF is shown in Figure 3. Endothermal process is observed during the first heating and is devoted to the water release of the BF. Nonetheless, around 150 °C the denaturalization of the collagen [29,30] and the 1st degradation step of the BF, observed in the TGA, are expected. A slight change of the peak related with the water is observed in the 1st heating, but the endothermal of the water evaporation is too big to account for it. Besides, a small change in the trend of the baseline is observed in the 2nd heating probably devoted to this degradation.

### 3.3. Effect of Leather Waste on Thermal Conductivity of Composites Materials

Thermal conductivity of neat HDPE and 20, 30, 40, and 50% BF composite specimen were analyzed and compared. The thermal conductivity values of dry BF waste specimen (per 0.2 g/cm^3^) were reported between 0.024–0.026 W/m °C in the literature [13]. The measure accounted for a thermal conductivity of 0.200 W/m °C for HDPE, and 0.219, 0.169, 0.207, and 0.190 W/m °C for 20%, 30%, 40%, and 50% BF, respectively. 

Incorporation of 20, 40, and 50% protein fraction in the HDPE matrix in the form of leather waste fibers provides comparable thermal resistance to neat HDPE. On the other hand, use of 30%BF improves thermal resistance for composite samples and provides a reduction of the thermal conductivity by 17% in comparison to that of neat HDPE samples. The reduction in the thermal conductivity is probably devoted to the discontinuity produced in the material by the BF fibers along with the insulation capacity of the leather BF. The thermal conductivity values of conventional heat insulating materials range from 0.034 to 0.173 W/m °C, (for example wool has 0.037 W/m °C and wood fibers 0.107 W/m °C) [13]. On the other hand, thermal conductivity required for pipeline applications, which is one of the most common industrial application areas of HDPE, reported as 0.43~0.52 W/m °C [31,32]. The value of the composite materials has the potential to be used as thermal insulator and in HDPE pipeline applications. 

### 3.4. FTIR

**Figure 4:**
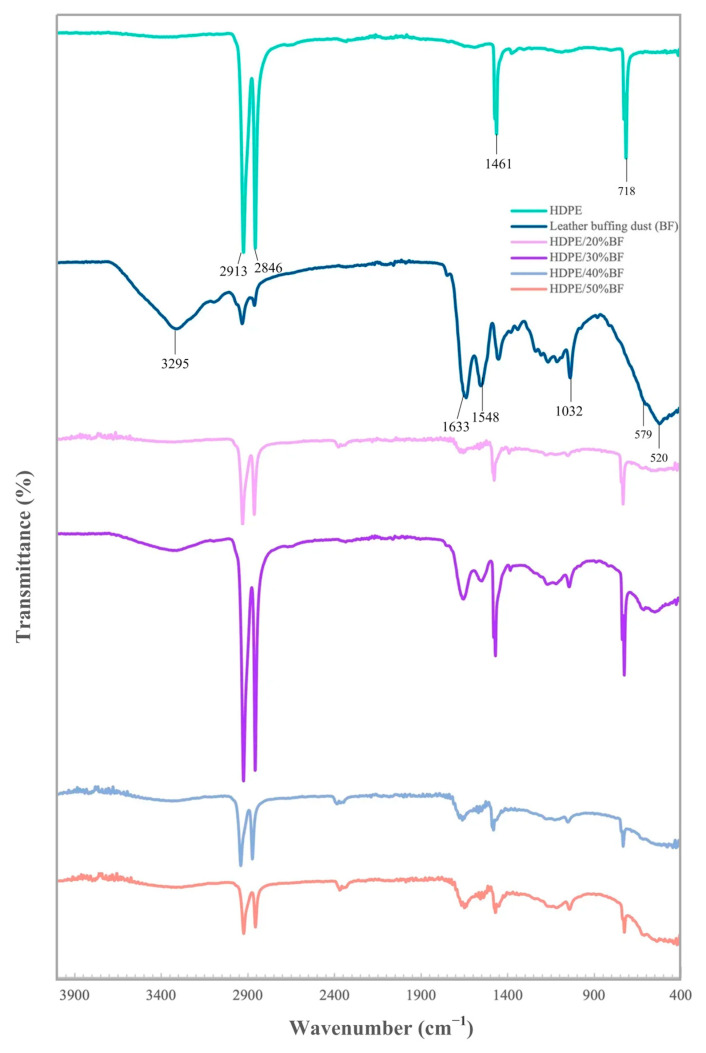
FTIR spectra of leather BF, HDPE and 20%, 30%, 40%, and 50% BF composite.

The FT-IR spectra of BF, neat HDPE, and BF/HDPE composites are given in Figure 4. HDPE shows 3 typical bands related to the symmetrical and asymmetrical CH_2_ rocking (720 cm^−1^), CH_2_ bending (1460–1470 cm^−1^) and CH_2_ stretching (2850–2920 cm^−1^) [33]. Thus, the other peaks in the composite materials must be devoted to BF. BF fibers showed the bands related with the aminoacids groups of collagen. The peak at 3295 cm^−1^ is assigned to the characteristic of –OH and –NH stretching vibration of amino acid in leather waste, and in the literature on this band reported between 3000 and 3500 cm^−1^ [11,12,34,35,36,37]. The bands comprised in the range 1600–1700 cm^−1^ and 1550–1570 cm^−1^ were related with amide I (C=O stretching of proteins) and amide II bands (C–N stretching and N–H bending of proteins) also to collagen respectively [34]. As previously described in the literature, similar results were reported for BF waste by Ambone et al (1625–1535 cm^−1^), Cardona et al. (1650–1540 cm^−1^), Senthil et al (1643–1540 cm^−1^), and for chromium shaving waste by Mohamed et al. (1650–1540 cm^−1^), Li et al (1645–1544 cm^−1^), and Liu et al (1647–1545 cm^−1^) [11,12,35,36,37,38]. 

The weak band in region 1000–1250 cm^−1^ could be attributed to –CN and –CO groups of amino acids [11,20,36,39]. Thus, the results confirmed the presence of quantitative protein content in BF waste. The peak at about 520–850 cm^−1^ accounts for Cr (III) species, which is in line with previously published data by, Liu et al (662–556 cm^−1^), Cardona et al (650 cm^−1^) and Senthil et al (852 cm^−1^) [12,35,38]. These bands were expected due to the Cr use in the leather tanning process. BF/HDPE composites showed all characteristic peaks of HDPE and BF. Of the bands at 2848–2915 cm^−1^, 1,461 cm^−1^, and 718 cm^−1^, all three are related to HDPE. Nonetheless, the characteristic absorption of amide groups of leather slightly shifts to lower wave number and changes from 1548–1536 cm^−1^ and to a higher wave number from 1633–1643 cm^−1^ compared with that of the original leather BF. These changes could indicate interfacial interactions between HDPE and leather BF, which improve their compatibility.

### 3.5. Chemical Resistance Test Results

**Figure 5:**
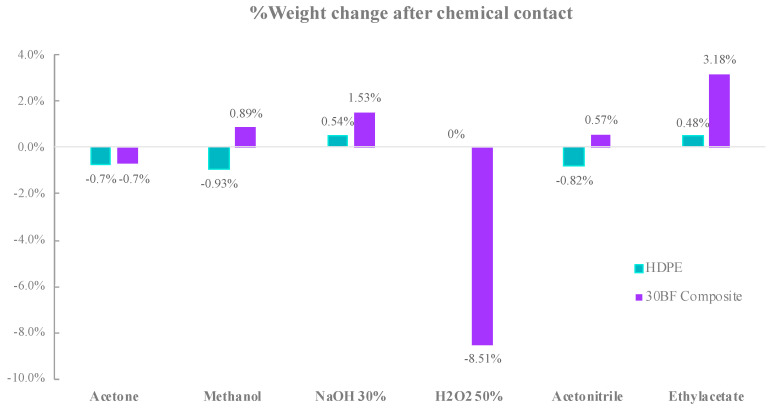
Relative weight change of HDPE and composite samples following 24 h of chemical contact.

Chemical resistance of neat HDPE and composites containing 30 wt% BF was investigated in terms of physical integrity and dissolution upon treatment with various chemicals. Following the treatment with chemicals, composites treated with hydrogen peroxide (H_2_O_2_ 50%) solution for 24 h, dissolved some of the leather fibers from composite samples, resulting in an approximate weight loss of 10% and changed physical integrity of samples (Figure 5). Different from other chemicals used in the experiment, hydrogen peroxide is a strong oxidizer, particularly in the concentrated state.

On the other hand, composites and neat HDPE samples did not exhibit any significant change both in visual appearance and weight loss in treatments with other chemicals such as acetone, methanol, NaOH, acetonitrile, and ethylacetate.

The oxidation state of chromium is an important indicator of toxicity and potential mobility. Chromium in the hexavalent state is highly toxic and soluble, whereas the trivalent state is much less toxic and relatively insoluble. Analysis of chromium leachate was performed to determine the mobility and the amount of extractable chromium in composites. Cr^3+^ in the leachate were found as 12 ppm for 30% BF composite and 21 ppm for 40% BF composites. The composite reinforced with a 30% BF provides comparable low levels in leachate to maximum allowed concentration MAC for nonhazardous waste (MAC = 10 mg/kg) [40] and previously published data regarding evolution of chromium oxide in natural rubber composites, subsequent to its exposure to different sanitizing chemicals (50 mg/kg) [41]. The strong interfacial bonding between polymer and leather waste, reported in our previously published paper, presumably prevented chromium leaching from composites [24]. The results for Cr(VI) suggested that the leachate contained Cr6+ at BDL (below detectable limit).

## 4. Conclusions

HDPE and animal-based natural fibers provided from leather industry waste were compounded in a lab scale internal mixer and processed through injection molding processes. The effect of leather fiber incorporation on thermal and structural properties of composites were studied in terms of TGA, DSC, and FTIR analysis. Chemical resistance and chromium-leaching properties of the composites were also investigated.

The experimental results showed that although the BF caused a slight reduction in thermal stability of the composite materials, composites filled with leather fibers from tannery waste can be processed without confronting a thermal degradation in the processing machines. The presence of the BF in composites does not have any impact on the melting process, and a negligible effect on crystallization process of composites due to slightly lower crystallization temperature. BF/HDPE composite showed all FT-IR characteristic peaks of HDPE and BF. On the other hand, incorporation of BF waste from chrome tanned leather into HDPE polymer matrix improved thermal insulation of final composite material by 17% due to thermal insulation capacity of leather fibers. Exposure of neat HDPE and composite samples to different chemicals did not exhibit any change in either visual appearance or weight of samples, except hydrogen peroxide (50%), which is a concentrated strong oxidizer. Further, the chromium leaching tests performed on composite samples provided comparable low leachate levels to maximum allowed concentration MAC for nonhazardous waste, and chromium VI is below detection limit in all the sample leachates.

The main advantages of BF/HDPE composites are use of animal-based natural fiber from industrial waste as inexpensive filler, and there is no need for previous treatments for preparing composite samples. The BF/HDPE composites could be a promising low-cost and sustainable alternative in HDPE applications, where high-mechanical strength and heat insulation is needed. Further, the obtained results bring to the light the suitability of HDPE for encapsulating BF, which prevents the Cr(VI) leaching.

## Figures and Tables

**Table 1:** Characteristics of buffing dust waste.

Tests	Values
Ash (wt%)	12.1
Chromium (III) (mg/g)	32
Fatty substances (wt%)	7.9
Nitrogen (wt%)	10.3
Protein (wt%)	57.5
Humidity (wt%)	50.9
pH	5.25
Length average (µm)	302.8
Diameter average (µm)	19.98

**Table 2:** Onset temperatures for 5%, 10%, 25%, and 50% of weight loss and residue at 700 °C.

Weight Loss (%)	HDPE	BF	HDPE + 20% BF	HDPE + 30% BF	HDPE + 40% BF	HDPE + 50% BF
5	423	281	340	319	289	317
10	440	306	403	364	333	341
25	458	342	454	449	430	433
50	469	408	472	471	468	471
Residue at 700 °C	2%	33%	7%	7.6%	12%	17%

**Table 3:** Thermal transition data of HDPE and HDPE/BF composites.

	HDPE	HDPE + 20% BF	HDPE + 30% BF	HDPE + 40% BF	HDPE + 50% BF
Crystallization temperature (°C)	117.2	116.4	117.9	117.4	114.2
Melting temperature (°C)	131.3	131.8	131.7	132.8	133.1
Enthalpy (J/g polymer)	210.09	207.1	209.3	167.4	208.1
Crystallinity (%)	71.7	70.7	71.4	57.1	71.0

## Data Availability

Data is available upon request to the corresponding author.

## Supplementary Materials

The following are available online at https://www.mdpi.com/article/10.3390/polym13193313/s1, Figure S1: DTGA of BF from 30 °C to 700 °C
