# Supplementary figures and images for: Valorization Strategy for Leather Waste as Filler for High-Density Polyethylene Composites: Analysis of the Thermal Stability, Insulation Properties and Chromium Leaching
Source: Polymers (Basel). 2021 Sep 28;13(19):3313. doi: 10.3390/polym13193313 (PMC8512770; doi:10.3390/polym13193313)

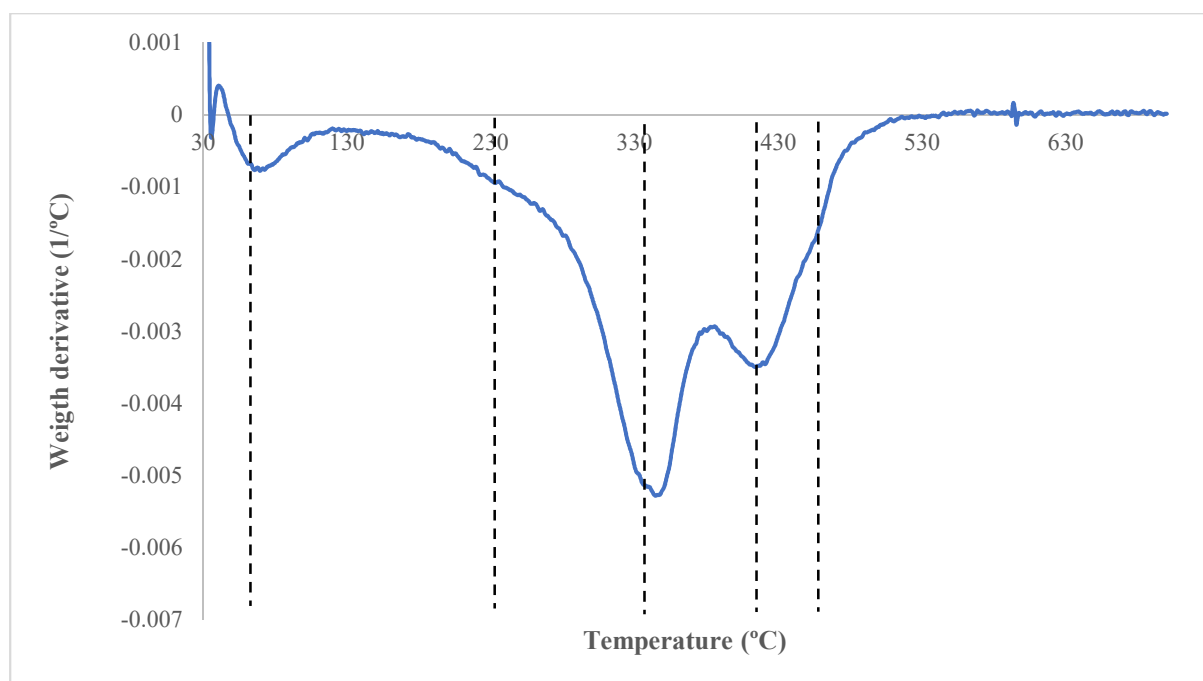

**Figure S1.** DTGA of BF from 30°C to 700°C

Supplement: Supplementary file 1 [file polymers-13-03313-s001.zip › polymers-1329139-supplementary.pdf]
